# Variation in Population Attributable Fraction of Dementia Associated With Potentially Modifiable Risk Factors by Race and Ethnicity in the US

**DOI:** 10.1001/jamanetworkopen.2022.19672

**Published:** 2022-07-06

**Authors:** Mark Lee, Eric Whitsel, Christy Avery, Timothy M. Hughes, Michael E. Griswold, Sanaz Sedaghat, Rebecca F. Gottesman, Thomas H. Mosley, Gerardo Heiss, Pamela L. Lutsey

**Affiliations:** 1 Department of Sociology, University of Minnesota, Minneapolis; 2 Minnesota Population Center, University of Minnesota, Minneapolis; 3 Department of Epidemiology, Gillings School of Global Public Health, University of North Carolina at Chapel Hill; 4 Department of Medicine, School of Medicine, University of North Carolina at Chapel Hill; 5 Department of Internal Medicine, Wake Forest School of Medicine, Winston-Salem, North Carolina; 6 Alzheimer’s Disease Research Center, Wake Forest School of Medicine, Winston-Salem, North Carolina; 7 School of Medicine, University of Mississippi Medical Center, Jackson; 8 Division of Epidemiology and Community Health, University of Minnesota School of Public Health, Minneapolis; 9 National Institute of Neurological Disorders and Stroke, National Institutes of Health, Bethesda, Maryland

## Abstract

**Importance:**

Identifying modifiable risk factors that are associated with dementia burden across racial and ethnic groups in the population can yield insights into the potential effectiveness of interventions in preventing dementia and reducing disparities.

**Objective:**

To calculate the population attributable fraction (PAF) of dementia associated with 12 established modifiable risk factors for all US adults, as well as separately by race and ethnicity.

**Design, Setting, and Participants:**

This cross-sectional study used survey data from nationally representative samples of US adults. PAFs were calculated using relative risks and prevalence estimates for 12 risk factors. Relative risks were taken from meta-analyses, as reported in a 2020 systematic review. Prevalence estimates for risk factors were derived from nationally representative cross-sectional survey data collected between 2011 and 2018. Combined PAFs were adjusted for risk factor communality using weights derived from the Atherosclerosis Risk in Communities (ARIC) study (1987-2018). Analyses were conducted May through October 2021.

**Exposures:**

Low education, hearing loss, traumatic brain injury, hypertension, excessive alcohol consumption, obesity, smoking, depression, social isolation, physical inactivity, diabetes, and air pollution.

**Main Outcomes and Measures:**

PAF for each dementia risk factor, a combined PAF, and the decrease in the number of prevalent dementia cases in 2020 that would be expected given a 15% proportional decrease in each exposure.

**Results:**

Among all US adults, an estimated 41.0% (95% CI, 22.7%-55.9%) of dementia cases were attributable to 12 risk factors. A 15% proportional decrease in each risk factor would reduce dementia prevalence in the population by an estimated 7.3% (95% CI, 3.7%-10.9%). The estimated PAF was greater for Black and Hispanic than it was for White and Asian individuals. The greatest attributable fraction of dementia cases was observed for hypertension (PAF, 20.2%; 95% CI, 6.3%-34.4%), obesity (PAF, 20.9%; 95% CI, 13.0%-28.8%), and physical inactivity (PAF, 20.1%; 95% CI, 9.1%-29.6%). These factors were also highest within each racial and ethnic group, although the proportions varied.

**Conclusions and Relevance:**

A large fraction of dementia cases in the US were associated with potentially modifiable risk factors, especially for Black and Hispanic individuals. Targeting and reducing these risk factors may curb the projected rise in dementia cases over the next several decades.

## Key Points

**Question:**

What proportion of dementia cases in the US are associated with established modifiable risk factors, and does this differ by race or ethnicity?

**Findings:**

In this cross-sectional study of risk factor prevalence estimates from 5 large, nationally representative surveys of US adults applied to risk ratio estimates drawn from meta-analyses, the estimated proportion of the population with dementia that was associated with modifiable factors was 41.0%; this varied by race and ethnicity: 35.8% for Asian American, 45.6% for Black, 46.7% for Hispanic, and 39.4% White individuals. The factors with the greatest contributions were hypertension, obesity, and physical inactivity.

**Meaning:**

These results suggest that reducing the prevalence of modifiable dementia risk factors may be associated with less racial and ethnic disparity and lower dementia rates among US adults.

## Teaser

This cross-sectional study assesses the population attributable fraction of dementia associated with 12 modifiable among US adults overall and by race and ethnicity.

## Introduction

As the US population ages, the number of people living with dementia is projected to rise markedly in coming decades—from approximately 5.8 million in 2020 to 13.8 million by 2050.^1^ In response to this looming epidemic, the federal government has set an ambitious goal to prevent and treat dementia by 2025.^2^ Because treatments for dementia are currently limited, reducing potentially modifiable dementia risk factors may be the most effective way to curb future dementia rates.^3,4^ Consequently, it is imperative to identify and target potentially modifiable risk factors that contribute to the dementia burden in the US population.

In the last decade, several studies have estimated the fraction of global dementia cases that are associated with potentially modifiable risk factors using population-based data.^3,4,5,6,7^ Most recently, a report from the Lancet Commission synthesized evidence from population-based observational studies and, when available, randomized clinical trials. The authors highlighted 12 risk factors with strong evidence of being causally related to dementia onset: low education, hearing loss, traumatic brain injury (TBI), hypertension, excessive alcohol consumption, obesity, smoking, depression, social isolation, physical inactivity, diabetes, and air pollution.^3^ Using risk ratios from meta-analyses and global prevalence estimates of each risk factor, the authors of the report estimated that up to 40% of dementia cases worldwide were associated with these 12 risk factors.

The most recent estimate of dementia fractions associated with potentially modifiable risk factors in the US was published in 2014 using prevalence estimates from 2010 or earlier.^6^ Since then, new risk factors for dementia have been identified, and the prevalence of previously known dementia risk factors has changed.^8,9,10,11^ Therefore, an update is needed. Drawing on evidence reviewed by the Lancet Commission, we calculated contemporary estimates of the fraction of dementia cases in the US that are associated with 12 potentially modifiable dementia risk factors. We calculated population attributable fractions (PAFs) for each risk factor alone and in combination with the others.

We also calculated novel estimates of dementia PAFs for separate racial and ethnic groups. To our knowledge, previous research has only considered the US population in aggregate.^5,6^ However, this approach may disguise intranational heterogeneity, as has been shown among ethnic groups in New Zealand.^12^ There are stark disparities in dementia rates across groups in the US.^13,14^ Compared with prevalence among non-Hispanic White individuals, the prevalence of dementia is twice as high among non-Hispanic Black and 1.5 times as high among Hispanic individuals.^15^ Identifying the primary drivers of dementia burden within different racial and ethnic groups is a critical precursor to crafting policies with an equitable health impact. To address these research gaps, the central questions driving this study were: (1) What proportion of dementia cases in the US are associated with potentially modifiable risk factors? and (2) Does this proportion vary by race and ethnicity?

## Methods

### Data Sources

The relative risk (RR) for dementia associated with each of the 12 risk factors was taken from the meta-analyses as reported by the Lancet Commission.^3^ Because a meta-analysis was not available for air pollution, the RR associated with this factor came from a single high-quality study.^16^ The Lancet Commission grouped risk factors according to the period within the life course during which exposure matters most for future dementia risk, as indicated by previous research.^3^ Education is an early life factor (for participants ages 45 years or younger) because most individuals complete their formal schooling in young adulthood; hearing loss, TBI, hypertension, excessive alcohol consumption, and obesity are classified as midlife factors (ages 45 to 64 years); smoking, depression, social isolation, physical inactivity, diabetes, and air pollution are classified as later life factors (ages 65 years and older). These RRs represent the most recent and highest-quality estimates of the relationship between each risk factor and dementia.

National and race-specific prevalence estimates of each risk factor (except air pollution) are based on recent data from 4 different cross-sectional surveys: the American Community Survey^17^; National Health and Nutrition Examination Survey^18^; National Health Interview Survey^19^; and the National Social Life, Health, and Aging Project.^20^ We used the provided sampling weights from each survey so that prevalence estimates would be representative of the US adult population. Race and ethnicity was self-reported in each data source, and categorized as non-Hispanic White (hereafter White), non-Hispanic Black (Black), non-Hispanic Asian (Asian), and Hispanic. To estimate exposure to air pollution, we combined census tract–level estimates of air quality from the Center for Air, Climate, and Energy Solutions^21^ with census tract–level population counts by race and ethnicity from the National Historical Geographic Information System.^22^ The definitions and data sources used to calculate the prevalence of each exposure are displayed in Table 1. To the extent possible, we applied the same definition of exposure as had been used in the RRs reported by the Lancet Commission.^3^ Further details on the operationalization of each risk factor are provided in the eMethods in the Supplement. The ARIC study protocols are approved by their local institutional review boards, and participants provided written informed consent. All data were deidentified and publicly available. This study followed the Strengthening the Reporting of Observational Studies in Epidemiology (STROBE) reporting guideline.

**Table 1.** Definitions of Dementia Risk Factors and Sources of Prevalence Estimates

Risk factor	Definition	Source (year)
Less education	Self-reported <12 y of schooling	ACS (2014-2018)
Obesity	Measured BMI ≥30	NHANES (2015-2018)
Hypertension	Self-report of taking antihypertensive medications or measured SBP ≥140 mm Hg or measured DBP ≥90 mm Hg	NHANES (2015-2018)
Diabetes	Self-report of diagnosis or measured fasting plasma glucose ≥126 mg/dL or measured HbA_1C_ ≥6.5%	NHANES (2015-2018)
Hearing loss	Pure-tone mean >25 dB hearing threshold in better ear measured at 0.5, 1, 2, and 4 kHz	NHANES (2015-2016)
Smoking	Self-report of current smoking	NHIS (2014-2018)
Physical inactivity	Self-report of not doing either 75 min/wk of vigorous activity or 150 min/wk of moderate activity or 150 min/wk of moderate/vigorous activity	NHIS (2014-2018)
Excessive alcohol consumption	Self-report of drinking more than 14 alcoholic drinks (eg, 12 oz of beer, 5 oz of wine, 1.5 oz of spirits) per wk	NHIS (2014-2018)
Social isolation	Self-report of contact with family, friends, religious organizations, organized groups, or volunteering less than once per mo	NSHAP (2015-2016)
Traumatic brain injury	Self-report of lifetime head injury resulting in loss of consciousness	NHANES (2011-2014)
Depression	PHQ-9 score ≥10	NHANES (2015-2018)
Air pollution	NO_2_ concentration ≥8.7 ppb at census tract level	CACES (2015)

Abbreviations: ACS, American Community Survey^17^; BMI, body mass index (calculated as weight in kilograms divided by height in meters squared); CACES, Center for Air, Climate, and Energy Solutions^21^; NHANES, National Health and Nutrition Examination Survey^18^; NHIS, National Health Interview Survey^19^; NSHAP, National Social Life Health and Aging Project^20^; PHQ-9, Patient Health Questionnaire-9.

We calculated prevalence for each risk factor within the age range suggested by the Lancet Commission.^3^ This is important because some factors could be influenced by cognitive status in late life (eg, weight loss among people with Alzheimer disease).^23^ Therefore, prevalence of low education included respondents aged 25 to 44 years; prevalence of hearing loss, TBI, hypertension, alcohol use disorder, and obesity included those aged 45 to 64 years; and prevalence of smoking, depression, physical inactivity, diabetes, and air pollution included respondents aged 65 years and older. We did not restrict the age range for national estimates of social isolation because this was drawn from a smaller sample (ie, respondents were aged 49 to 95 years).

To estimate the combined prevalence of dementia associated with all 12 risk factors, we adjusted for the communality of risk factors (using methods described in Statistical Analysis). We relied on population-based data from a sample of Black and White individuals in the Atherosclerosis Risk in Communities (ARIC) study (15 792 participants) to calculate communality weights. The ARIC data are well-suited for this task because they contain measures of each risk factor in a large, population-based, racially diverse sample. However, there were some small differences in how the exposure was measured between ARIC and the national data (eMethods in the Supplement).

### Statistical Analysis

We estimated the unadjusted PAF for each risk factor, *e*, using Levin’s formula^24^: PAF_e_ = P_e_(RR_e_ − 1)/(P_e_[RR_e_ − 1] + 1), where P_e_ is the population prevalence of risk factor *e* and RR*e* is the relative risk of dementia associated with exposure to risk factor *e*. To calculate PAFs for different racial and ethnic groups, we used the RR reported in the Lancet Commission report^3^ combined with group-specific prevalence estimates of exposure from nationally representative data.

Because risk factors co-occur within individuals, summing the PAFs of each risk factor would yield an inflated estimate of their combined contribution to the population burden of dementia. Following the method introduced by Norton et al,^6^ we used communality weights to adjust for risk factor overlap among participants. First, using ARIC data, we calculated the pairwise tetrachoric correlation between all 12 risk factors. Next, we conducted a principal components analysis on the tetrachoric correlation matrix. The communality for each risk factor is equal to the sums of squares of the loadings in all principal components with an eigenvector greater than 1. We then weighted each risk factor using the formula^6^: W_e_ = 1 − communality_e_. Next, we calculated the combined adjusted PAF using the formula^6^: PAF = 1 − [(1 − W_1_*PAF_1_)(1 − W_2_*PAF_2_)(1 − W_3_*PAF_3_)…]. Finally, we estimated the adjusted PAF for each individual risk factor using the formula introduced in the 2017 Lancet Commission report^4^: adjusted PAF_e_ = ([PAF_e_ / ∑ PAF_e_] * combined PAF).

The PAF estimates the maximum fraction of dementia cases that could potentially be prevented by eliminating risk factors from a population. However, 100% reduction in risk factors is not feasible. The Risk Reduction Subcommittee of the National Alzheimer Project Act Advisory Council recently set a goal of reducing dementia risk factors 15% by 2030.^25^ To estimate how this goal may affect the number of prevalent dementia cases in the US, we calculated how many fewer prevalent dementia cases would be expected given a 15% proportional reduction in each risk factor. First, we calculated the potential impact fraction (PIF) associated with a reduction in each risk factor using the formula^26^: PIF_e_ = ([P_e_ − P^’^_e_] * [RR_e_ − 1])/(P_e_[RR_e_ − 1] + 1), where P_e_ is the observed prevalence of risk factor *e* in the population, P^’^_e_ is the counterfactual prevalence of that risk factor following a 15% proportional reduction, and RR_e_ is the relative risk of dementia associated with risk factor *e*. We then estimated the PIF associated with a 15% proportional reduction in all risk factors combined using an adaptation of the combined PAF formula: PIF = 1-[(1 − W_1_*PIF_1_)(1 − W_2_*PIF_2_)(1 − W_3_*PIF_3_)…]. Finally, we estimated how many fewer dementia cases (rounded to the nearest 100) in the US in 2020 would be expected in this counterfactual scenario by multiplying the PIFs by the estimated number of dementia cases in the population in 2020.^27^ We completed these calculations both for the total US adult population and separately by race and ethnicity. Following previous studies, we used confidence intervals from the RR for each risk factor to calculate upper and lower bounds for estimates of PAFs, PIFs, and the number of dementia cases prevented for each risk factor.^5,6^

## Results

Estimates of risk factor prevalence were derived from 5 large, nationally representative data sources with roughly equal numbers of men and women and, in some cases, oversamples of racial and ethnic minority groups (Table 1). Communality ranged from 30.0% for physical inactivity to 85.4% for TBI (higher communality indicates greater correlation with other risk factors (Table 2). Among the total US population, higher prevalence of dementia cases were associated with midlife hypertension (20.2%; 95% CI, 6.3%-34.4%), midlife obesity (20.9%; 95% CI, 13.0%-28.8%), and late life physical inactivity (20.1%; 95% CI, 9.1%-29.6%). Taking all 12 risk factors into account, and weighting by communality, 41.0% (95% CI, 22.7%-55.9%) of dementia cases in the US population were associated with these potentially modifiable risk factors (Table 3).

**Table 2.** Risk Factor Relative Risk and Communality

Risk factor	RR (95% CI)^a^	Communality, %^b^
Less education	1.59 (1.26-2.01)	72.0
Hearing loss	1.94 (1.38-2.73)	83.5
TBI	1.84 (1.54-2.20)	85.4
Hypertension	1.61 (1.16-2.24)	53.4
Excessive alcohol	1.18 (1.06-1.31)	59.8
Obesity	1.60 (1.34-1.92)	65.9
Smoking	1.59 (1.15-2.20)	63.5
Depression	1.90 (1.55-2.33)	56.6
Social isolation	1.57 (1.32-1.85)	43.3
Physical inactivity	1.39 (1.16-1.67)	30.0
Diabetes	1.54 (1.33-1.79)	54.5
Air pollution	1.09 (1.07-1.11)	63.8

Abbreviations: ARIC, Atherosclerosis Risk in Communities; PCA, principal components analysis; RR, relative risks; TBI, traumatic brain injury.

^a^ Relative risks plus confidence intervals for each risk factor come from recent meta-analyses, as reported in the Lancet Commission.^3^

^b^ Communality is calculated using principal components analysis for ARIC data. The PCA identified 5 components with eigenvalues greater than one that together explained 61% of the variation among the 12 risk factors. Higher communality percentages indicate greater correlation with other risk factors. See Methods for more details.

**Table 3.** Dementia Risk Factor Prevalences and Population Attributable Fractions Among US Adults, Overall and by Race and Ethnicity

Risk factor	Total population		Hispanic		Non-Hispanic Asian		Non-Hispanic Black		Non-Hispanic White	
	Prevalence, %	PAF, % (95% CI)	Prevalence, %	PAF, % (95% CI)	Prevalence, %	PAF, % (95% CI)	Prevalence, %	PAF, % (95% CI)	Prevalence, %	PAF, % (95% CI)
Less education	10.7	6.0 (2.7-9.8)	27.1	14.0% (6.6-21.5)	6.4	3.7 (1.6-6.1)	10.6	6.0% (2.7-9.7)	5.5	3.2 (1.4-5.3)
Hearing loss	10.8	8.9 (3.9-15.7)	13.1	10.5 (4.7-18.5)	6.9	5.8 (2.6-10.7)	6.5	5.5 (2.4-10.1)	10.6	8.7 (3.9-15.7)
TBI	17.1	12.0 (8.5-17.0)	10.3	7.6 (5.3-11.0)	6.0	4.6 (3.1-6.7)	9.2	6.9 (4.7-9.9)	20.1	13.9 (9.8-19.4)
Hypertension	42.2	20.2 (6.3-34.4)	38.5	18.8 (5.8-32.3)	38.5	18.8 (5.8-32.3)	61.0	26.8 (8.9-43.1)	39.8	19.3 (6.0-33.0)
Excessive alcohol	3.6	0.7 (0.2-1.1)	2.0	0.4 (0.1-0.6)	0.7	0.1 (0.0-0.2)	2.7	0.5 (0.2-0.8)	4.2	0.8 (0.3-1.3)
Obesity	44.0	20.9 (13.0-28.8)	48.3	22.5 (14.1-30.8)	14.6	8.1 (4.7-11.8)	54.3	24.6 (15.6-33.3)	43.5	20.7 (12.9-28.6)
Smoking	8.5	4.9 (1.3-9.3)	6.9	4.0 (1.0-7.6)	4.9	2.9 (0.7-5.6)	11.7	6.6 (1.7-12.3)	8.4	4.8 (1.1-9.2)
Depression	7.4	6.2 (3.9-9.0)	10.7	8.8 (5.6-12.5)	4.3	3.7 (2.3-5.4)	6.6	5.6 (3.5-8.1)	7.2	6.1 (3.8-8.7)
Social isolation	11.9	6.7 (3.7-9.2)	24.0	12.6 (7.1-16.9)	8.0	4.6 (2.5-6.4)	12.1	6.8 (3.7-9.3)	10.8	6.1 (3.3-8.4)
Physical inactivity	62.8	20.1 (9.1-29.6)	68.6	21.5 (9.9-31.5)	56.6	18.5 (8.3-27.5)	73.2	22.6 (10.5-32.9)	61.3	19.7 (8.9-29.1)
Diabetes	28.6	12.5 (8.6-18.4)	41.0	17.0 (11.9-24.5)	44.1	18.1 (12.7-25.8)	37.2	15.7 (10.9-22.7)	25.4	11.3 (7.7-16.7)
Air pollution	22.8	2.2 (1.6-2.4)	44.4	4.3 (3.0-4.7)	55.2	5.2 (3.7-5.7)	41.3	4.0 (2.8-4.3)	17.2	1.7 (1.2-1.9)
Combined factors^a^	NA	41.0 (22.7-55.9)	NA	46.7 (27.3-61.5)	NA	35.8 (19.5-49.9)	NA	45.6 (25.7-60.5)	NA	39.4 (22.7-55.9)

Abbreviations: NA, not applicable; PAF, population attributable fraction; TBI, traumatic brain injury.

^a^ Combined PAF estimate is weighted by risk factor communality. See Methods section for details.

There were racial and ethnic differences in the prevalence and PAFs for dementia associated with each risk factor (Table 3). For example, 5.5% of White adults aged 25 to 44 years had fewer than 12 years of education compared with 27.1% of Hispanic individuals. Consequently, the PAF for dementia associated with low education was higher for Hispanic (14.0%; 95% CI, 6.6%-21.5%) than White individuals (3.2%; 95% CI, 1.4%-5.3%). However, White adults aged 45 to 64 years reported TBI at higher rates than other groups. When all risk factors are combined, they were estimated to be associated with approximately 39.4% (95% CI, 22.7%-55.9%) of dementia cases among White, 45.6% (95% CI, 25.7%-60.5%) among Black, 46.7% (95% CI, 27.3%-61.5%) among Hispanic, and 35.8% (95% CI, 19.5%-49.9%) among Asian individuals.

Hypertension, obesity, and physical inactivity were associated with the greatest fraction of attributable dementia cases in each racial and ethnic group (Figure). For example, the unweighted PAF for hypertension was 20.2% (95% CI, 6.3%-34.4%), while the weighted PAF was 6.8% (95% CI, 2.3%-10.4%). The only exception was among Asian individuals, for whom the obesity PAF was modest. Second, as expected, the weighted PAFs for each risk factor were substantially smaller than the unweighted PAFs. The unweighted PAFs assumed that each risk factor could be altered in isolation, while the weighted PAFs accounted for the communality across risk factors.

**Figure.**
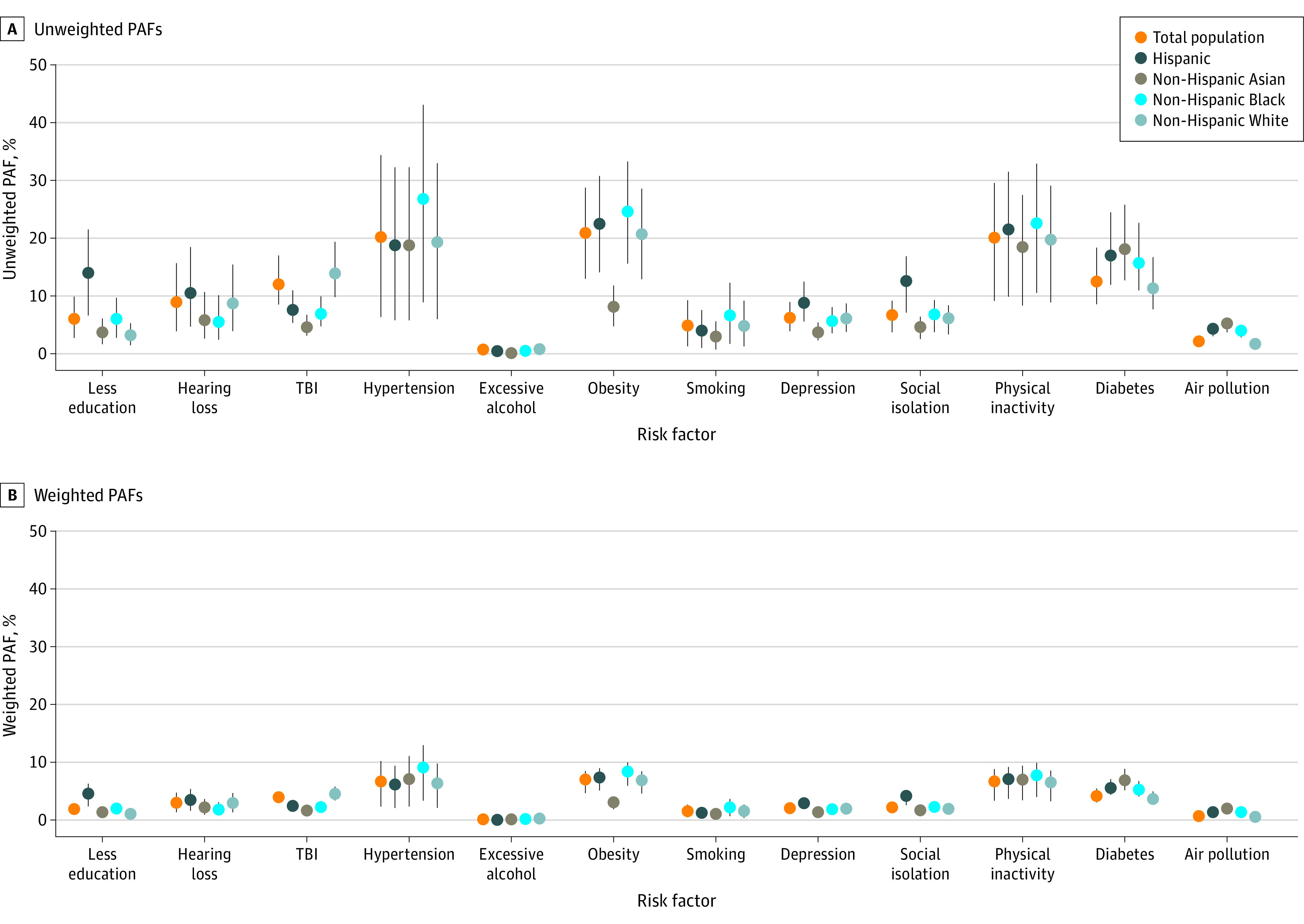
Unweighted and Weighted Populations Attributable Fractions (PAFs) for Each Risk Factor by Race and Ethnicity Weighted PAFs account for the communality (correlation) between risk factors. See Methods for details.

Among all US adults, there were an estimated 5 814 000 prevalent dementia cases in 2020.^27^ However, reductions in risk factor prevalence could have prevented tens of thousands of cases. A 15% reduction in obesity prevalence (ie, from 44.0% to 37.4%), for example, would be associated with approximately 3.1% (95% CI, 2.0%-4.3%) lower dementia prevalence, which would correspond with approximately 182 100 (95% CI, 113 500-251 300) cases in 2020 (Table 4). A 15% reduction in all risk factors combined would be associated with 7.3% (95% CI, 3.7%-10.9%) lower prevalence, corresponding to 427 000 (95% CI, 216 600-636 300) cases in 2020.

**Table 4.** PIF and Total Estimated Reductions in US Dementia Cases Expected With a 15% Proportional Risk Factor Reduction in 2020 by Race and Ethnicity^a^

Risk factor	Total population		Hispanic		Non-Hispanic Asian		Non-Hispanic Black		Non-Hispanic White	
	PIF, % (95% CI)	Fewer cases, No. (95% CI)^b^	PIF, % (95% CI)	Fewer cases, No. (95% CI)^b^	PIF, % (95% CI)	Fewer cases, No. (95% CI)^b^	PIF, % (95% CI)	Fewer cases, No. (95% CI)^b^	PIF, % (95% CI)	Fewer cases, No. (95% CI)^b^
Less education	0.9 (0.4-1.5)	52 600 (23 600-85 100)	2.1 (1.0-3.2)	12 500 (5900-19 100)	0.6 (0.2-0.9)	1200 (500-1900)	0.9 (0.4-1.5)	6500 (2900-10 500)	0.5 (0.2-0.8)	20 100 (8900-33 000)
Hearing loss	1.3 (0.6-2.4)	77 300 (34 400-137 300)	1.6 (0.7-2.8)	9400 (4200-16 500)	0.9 (0.4-1.6)	1900 (800-3400)	0.8 (0.4-1.5)	6000 (2600-11 000)	1.3 (0.6-2.3)	54 700 (24 300-97 300)
TBI	1.8 (1.3-2.6)	104 900 (73 700-148 500)	1.1 (0.8-1.7)	6800 (4700-9800)	0.7 (0.5-1.0)	1500 (1000-2100)	1.0 (0.7-1.5)	7500 (5200-10 800)	2.1 (1.5-2.9)	87 000 (61 500-122 000)
Hypertension	3.0 (0.9-5.2)	176 200 (55 200-299 600)	2.8 (0.9-4.8)	16 700 (5200-28 200)	2.8 (0.9-4.8)	6000 (1800-10 300)	4.0 (1.3-6.5)	29 200 (9700-46 900)	2.9 (0.9-5.0)	121 000 (37 600-207 500)
Excessive alcohol	0.1 (0.0-0.2)	6200 (1900-9600)	0.1 (0.0-0.1)	400 (100-500)	0.0 (0.0-0.0)	0 (0-100)	0.1 (0.0-0.1)	600 (200-900)	0.1 (0.0-0.2)	5200 (1600-8100)
Obesity	3.1 (2.0-4.3)	182 100 (113 500-251 300)	3.4 (2.1-4.6)	20 000 (12 600-27 400)	1.2 (0.7-1.8)	2600 (1500-3800)	3.7 (2.3-5.0)	26 800 (17 000-36 300)	3.1 (1.9-4.3)	130 000 (80 900-179 500)
Smoking	0.7 (0.2-1.4)	42 300 (11 000-80 700)	0.6 (0.2-1.1)	3500 (900-6800)	0.4 (0.1-0.8)	900 (200-1800)	1.0 (0.3-1.8)	7100 (1900-13 400)	0.7 (0.2-1.4)	30 100 (7800-57 500)
Depression	0.9 (0.6-1.3)	54 500 (34 100-78 100)	1.3 (0.8-1.9)	7800 (5000-11 100)	0.6 (0.3-0.8)	1200 (700-1700)	0.8 (0.5-1.2)	6100 (3800-8800)	0.9 (0.6-1.3)	38 200 (23 900-54 900)
Social isolation	1.0 (0.6-1.4)	58 100 (32 000-80 100)	1.9 (1.1-2.5)	11 200 (6400-15 100)	0.7 (0.4-1.0)	1500 (800-2000)	1.0 (0.6-1.4)	7400 (4100-10 200	0.9 (0.5-1.3)	38 200 (21 000-52 800)
Physical inactivity	3.0 (1.4-4.4)	175 100 (79 600-258 300)	3.2 (1.5-4.7)	19 200 (8800-28 100)	2.8 (1.2-4.1)	5900 (2600-8700)	3.4 (1.6-4.9)	24 700 (11 400-35 800)	3.0 (1.3-4.4)	123 600 (56 100-182 800)
Diabetes	1.9 (1.3-2.8)	109 100 (75 200-160 700)	2.6 (1.8-3.7)	15 200 (10 600-21 800)	2.7 (1.9-3.9)	5700 (4000-8200)	2.4 (1.6-3.4)	17 100 (11 900-24 700)	1.7 (1.2-2.5)	70 800 (48 600-104 900)
Air pollution	0.3 (0.2-0.4)	19 400 (13 700-21 300)	0.6 (0.5-0.7)	3800 (2700-4100)	0.8 (0.6-0.9)	1700 (1200-1800)	0.6 (0.4-0.7)	4300 (3100-4700)	0.3 (0.2-0.3)	10 600 (7500-11 700)
Combined factors^c^	7.3 (3.7-10.9)	427 000 (216 600-636 300)	8.7 (4.6-12.6)	51 500 (27 300-75 000)	6.2 (3.2-9.3)	13 200 (6700-19 800)	8.4 (4.3-12.2)	60 700 (31 000-88 600)	7.0 (3.5-10.5)	293 000 (148 100-438 400)

Abbreviations: PIF, potential impact fraction; TBI, traumatic brain injury.

^a^ A 15% proportional reduction is based on the proportion of US adults in each racial or ethnic group that would be considered exposed to each risk factor after a hypothetical intervention that reduced the fraction in the high-risk category by three-twentieths (eg, moving from 22.8% of individuals living in census tracts with NO_2_ exposure 8.7 ppb or greater to 19.4% by improving air quality).

^b^ Number of cases based on recent published estimates of 5 814 000 prevalent dementia cases among all US adults, 4 186 000 prevalent cases among White, 726 000 prevalent cases among Black, 594 000 prevalent cases among Hispanic, and 212 000 prevalent cases among Asian individuals in 2020.^27^

^c^ Combined estimate is weighted by risk factor communality. See the Methods section for details.

There were racial and ethnic differences in the PIF, evident in the differences in the observed prevalence of each risk factor. A 15% reduction in all risk factors was associated with a 7.0% (95% CI, 3.5%-10.5%) decrease in dementia among White (293 000 cases; 95% CI, 148 100-438 400 cases), 8.4% (95% CI, 4.3%-12.2%) among Black (60 700 cases; 95% CI, 31 000-88 600 cases), 8.7% (95% CI, 4.6%-12.6%) among Hispanic (51 500 cases; 95% CI, 27 300-75 000 cases), and 6.2% (95% CI, 3.2%-9.3%) among Asian individuals (13 200 cases; 95% CI, 6700-19 800 cases).

## Discussion

In this study, we found that 41% of dementia cases in the US were associated with 12 potentially modifiable risk factors. The Risk Reduction Subcommittee of the National Alzheimer Project Act Advisory Council set a goal of reducing dementia risk factors by 15%.^25^ We estimate that a 15% proportional reduction in risk factor prevalence would be associated with approximately 427 000 fewer prevalent dementia cases in the US population based on data from 2020. Although the PAF we estimate in the US closely matches the global estimate from a recent Lancet Commission report,^3^ the risk factors most commonly associated with a greater dementia burden differ. Globally, hearing loss, less education, and smoking contribute most to dementia rates. In the US, where the risk factor profile of the population differs, the largest fractions of dementia cases were associated with midlife hypertension, midlife obesity, and late life physical inactivity.

We calculated novel PAF estimates of dementia within racial and ethnic groups to assess the degree of intranational heterogeneity. The proportion of potentially preventable dementia cases was considerably higher among Black and Hispanic individuals than it was among White and Asian individuals. This indicates that modifiable determinants of dementia are particularly important for shaping rates of disease among Black and Hispanic individuals, who currently have substantially higher dementia prevalence than those who are White.^15^ However, there were notable similarities across groups in the risk factors most strongly associated with dementia burden. Interventions that effectively reduce hypertension, obesity, and physical inactivity (eg, by identifying and removing structural barriers to health among Black and Hispanic communities) may have an advantageous impact on dementia rates across the population.

### Strengths and Limitations

The data and methods used in this study have several strengths. The estimated RRs for each risk factor come from recent meta-analyses and represent the best available evidence.^3^ Although these RRs did not mutually adjust for all other risk factors, we accounted for the nonindependence of risk factors using communality weights, which is a conservative approach for estimating the combined PAF.^3,4,6^ While previous studies calculated communality weights using a sample of adults from the United Kingdom, we calculated weights using a diverse sample of US adults, which was more appropriate for this study. Prevalence estimates for the risk factors came from a total of 5 large, nationally representative data sets that provided the most rigorously quantified information available and are frequently used to study racial and ethnic differences in the US.

This study had several limitations. PAF estimates assume a causal relationship between each risk factor and dementia, and that the RR from meta-analyses is a good approximation of the true causal effect. If this assumption is incorrect, our PAF estimates may be higher or lower than the true value. While the body of evidence underlying each risk factor is robust, the relative risk estimates were derived from observational data.^3^ More work is needed to test whether intervening on these exposures lowers dementia risk. Results from the Finnish Geriatric Intervention Study to Prevent Cognitive Impairment and Disability (FINGER) showed that older adults benefited from a multidomain intervention (affecting diet, physical activity, cognitive training, and vascular risk management).^28^ However, results from other clinical trials have been somewhat mixed.^29^ Other causal relationships, such as the effect of educational attainment on dementia risk, are difficult to test using randomized trials. However, evidence from natural experiments—such as changes in mandatory schooling laws—are supportive of a causal effect.^30^

In most cases, the data allowed separate estimation for White, Black, Hispanic, and Asian individuals. Unfortunately, for social isolation, no data sources were available that provided estimates for Asian individuals. Therefore, we used prevalence estimates from all non-White, non-Black, and non-Hispanic respondents to approximate prevalence among Asian individuals for this risk factor. It is possible that this estimate is biased by the inclusion of other ethnic groups.

Levin’s PAF formula was intended for use with an unadjusted RR from a single risk factor and outcome.^31,32^ Although alternative methods have been developed for calculating PAFs using adjusted RRs and multiple risk factors, we were not able to use these methods because they require raw data.^33^ We accounted for risk factor overlap in the combined PAF estimate using communality weights, which discount the contribution of individual risk factors according to how strongly correlated they are with other risk factors. While this approach is limited, it was the only method available to us given the constraints of our data.

The relative risks were not estimated in each racial and ethnic group separately. However, previous research suggests that race does not significantly modify the association between key risk factors and dementia.^34^ Additionally, this analysis evaluated racial and ethnic disparities in risk factors, but it is masked to structural factors that put some groups at “risk of risks,” such as residential segregation, discrimination, and incarceration. Future analyses should investigate how these macro-level factors drive inequalities in dementia rates.

## Conclusions

Until an effective treatment for dementia is developed, delaying dementia onset in the population by targeting modifiable risk factors is the best tool to curb the projected increase in dementia cases. Policy makers should prioritize efforts to reduce the prevalence of midlife obesity, midlife hypertension, and late-life physical inactivity, which are currently associated with the largest fraction of dementia cases in the US. An important role also exists for national policies that, although not primarily intended to reduce dementia risk, may yield lower exposure to dementia risk factors (eg, policies related to education and environmental regulation). Continued efforts should be made to reduce racial and ethnic disparities in the 12 risk factors considered in this study, including addressing structural factors which underlie risk factor differences. Doing so could help to achieve cognitive health equity among America’s rapidly diversifying population of older adults.

## Notes

Supplement.
eMethods.
