# Supplementary material for: Variation in Population Attributable Fraction of Dementia Associated With Potentially Modifiable Risk Factors by Race and Ethnicity in the US
Source: JAMA Netw Open. 2022 Jul 6;5(7):e2219672. doi: 10.1001/jamanetworkopen.2022.19672 (PMC9260480; doi:10.1001/jamanetworkopen.2022.19672)
Supplement: Supplement. — eMethods. [file jamanetwopen-e2219672-s001.pdf]

Supplemental Online Content

Lee M, Whitsel E, Avery C, et al. Variation in population attributable fraction of dementia associated with potentially modifiable risk factors by race and ethnicity in the US. *JAMA Netw Open.* 2022;5(7):e2219672. doi:10.1001/jamanetworkopen.2022.19672

eMethods.

This supplemental material has been provided by the authors to give readers additional information about their work.

© 2022 Lee M et al. *JAMA Network Open.*

eMethods

1. Defining Risk Factors for Prevalence Estimates using Nationally Representative Cross-sectional Survey Data

Obesity, hypertension, and diabetes were assessed by trained technicians during a physical examination. Education, hearing loss, smoking, and physical activity (expressed as minutes of moderate and vigorous exercise per week) were self-reported. Excessive alcohol consumption was defined as reporting more than 14 drinks per week on average. Following the *Lancet* Commission,[1] social isolation was defined as having less than monthly contact with family, friends, religious organizations, social organizations, and volunteer groups. Lifetime TBI was measured in NHANES as self-report of a head injury at any age resulting in loss of consciousness. Depression was defined as a score of 10 or greater on the Patient Health Questionnaire (PHQ-9).[2] Based on evidence from a Canadian study[3] that was used to generate the RR for air pollution in the *Lancet* Commission report,[1] exposure to air pollution was defined as living in a census tract with NO_2 concentration equal to or greater than 8.7 parts per billion. Pollution data is only available for the 48 contiguous states, which comprise 99.3% of the American population.

To improve precision of race-specific prevalence estimates, we combined data from consecutive survey years to generate larger samples when possible. Race-specific estimates of air pollution exposure were generated by combining census-tract level information on air quality with census-tract level population estimates by race and Hispanic origin.[4]

2. Defining Risk Factors for Communality Estimates using the Atherosclerosis Risk in Communities Study.

Low education was defined as less than a high school diploma. Hearing loss was assessed during an audiometric examination and was defined as Pure-Tone Average ≥25 decibels hearing level in the better ear.[5] TBI was defined as self-report of head injury causing loss of consciousness. Hypertension was defined as systolic blood pressure > 140 mm Hg or diastolic blood pressure > 90 mm Hg or report of taking antihypertensive medication. Excessive alcohol consumption in ARIC was defined as greater than 14 drinks per week. Obesity was defined as body mass index ≥ 30 kg/m^2 using measured height and weight. Smoking was defined as current smoking. Depression in ARIC was measured using validated thresholds of the Center for Epidemiological Studies-Depression (CES-D) scale.[6] Social activities were not measured in ARIC, so isolation was defined as living without a spouse or partner. Physical inactivity was defined as the lowest quartile on a scale combining activity type (light, moderate, or vigorous), activity hours per week, and activity months per year. Diabetes was defined as fasting glucose ≥ 126 mg/dL or non-fasting glucose ≥ 200 mg/dL or using medication for diabetes or self-report of diabetes diagnosis. Exposure to air pollution in ARIC was defined as the 25th percentile or above for mean annual NO_2, which was the same percentile threshold used by the *Lancet* Commission to define exposure.[1] The observed prevalence of dementia risk factors in ARIC is displayed in eTable 1. The observed pairwise tetrachoric correlations between risk factors in ARIC is displayed in eTable 2.

eTable 1. Dementia risk factor prevalence: Atherosclerosis Risk in Communities study.

**Risk factor**	**Prevalence, %**
Low education	23.9
Hearing loss	67.6
TBI	14.8
Hypertension	35.0
Excessive alcohol	3.8
Obesity	27.6
Smoking	5.9
Depression	7.1
Social isolation	24.2
Physical inactivity	31.1
Diabetes	33.9
Air pollution	75.0

© 2022 Lee M et al. *JAMA Network Open.*

eTable 2. Pairwise tetrachoric correlations among dementia risk factors: Atherosclerosis Risk in Communities study.

Risk factor	1	2	3	4	5	6	7	8	9	10	11	12
1. Low education	1											
2. Hearing loss	0.11	1										
3. TBI	-0.15	0.09	1									
4. Hypertension	0.25	-0.02	-0.16	1								
5. Excessive alcohol	-0.06	0.09	0.09	0.05	1							
6. Obesity	0.18	0.01	-0.03	0.35	-0.12	1						
7. Smoking	0.10	0.03	-0.01	-0.10	0.21	-0.15	1					
8. Depression	0.31	0.00	0.04	0.17	0.03	0.20	0.15	1				
9. Social isolation	0.24	-0.09	-0.11	0.11	-0.03	0.13	0.10	0.21	1			
10. Physical inactivity	0.16	0.05	-0.08	0.14	-0.02	0.21	0.09	0.21	0.07	1		
11. Diabetes	0.22	0.01	-0.02	0.29	0.00	0.43	-0.05	0.20	0.08	0.15	1	
12. Air pollution	0.52	-0.08	-0.13	0.21	-0.10	0.12	-0.01	0.29	0.23	0.09	0.20	1

References

1. Livingston G, Huntley J, Sommerlad A, et al. Dementia prevention, intervention, and care: 2020 report of the Lancet Commission. *The Lancet*. 2020;396(10248):413-446. doi:10.1016/S0140-6736(20)30367-6

2. Kroenke K, Spitzer RL, Williams JBW. The PHQ-9. *J Gen Intern Med*. 2001;16(9):606-613. doi:https://doi.org/10.1046/j.1525-1497.2001.016009606.x

3. Chen H, Kwong JC, Copes R, et al. Exposure to ambient air pollution and the incidence of dementia: A population-based cohort study. *Environ Int*. 2017;108:271-277. doi:10.1016/j.envint.2017.08.020

4. Manson S, Schroeder J, Van Riper D, Kugler T, Ruggles S. *IPUMS National Historical Geographic Information System: Version 16.0 [Dataset].* IPUMS; 2021. http://doi.org/10.18128/D050.V16.0

5. Shukla A, Reed NS, Armstrong NM, Lin FR, Deal JA, Goman AM. Hearing Loss, Hearing Aid Use, and Depressive Symptoms in Older Adults—Findings from the Atherosclerosis Risk in Communities Neurocognitive Study (ARIC-NCS). *J Gerontol Ser B*. 2021;76(3):518-523. doi:10.1093/geronb/gbz128

6. Radloff LS. The CES-D Scale: A Self-Report Depression Scale for Research in the General Population. *Appl Psychol Meas*. 1977;1(3):385-401. doi:10.1177/014662167700100306

© 2022 Lee M et al. *JAMA Network Open.*
